# 

**Published:** 1923-03

**Authors:** 


					CONTENTS.
Page
Diagnosis of Peptic Ulcer and its Bearings on Treatment.
% T. Carwardine, M.S.
7i
Unsuccessful Tonsil and Adenoid Operat
ions.
By A. J. M. Wright,
MB, F.R.C.S. ...
84
^'Sht Ether Anaesthesia.
By A. L. Flemming, M.B., B.Ch.
ntracranial Tumours and the Occurrence of Papilloedema.
% A. E Iles, O.B.E., M B. Lond., F.R.C.S.
94
^rogress of the Medical Sciences
Psychiatry.
J. O. Symes, M.D. Lond.
fcviews of Books
*Qg,
Editorial Notes
Library of the Bristol Medico-Chirurgical Sooety
V1
"5
?Ca' Medical Notes  J (J N 4 ~ -1

				

